# The Emerging Role of the DDAH Proteins in Psychiatric Disorders

**DOI:** 10.1016/j.bpsgos.2025.100574

**Published:** 2025-07-28

**Authors:** Magdalini R. Vareltzoglou, Roman N. Rodionov, Anthony C. Vernon, Nadine Bernhardt

**Affiliations:** a Department of Psychiatry and Psychotherapy, University Hospital Carl Gustav Carus, Dresden University of Technology, Dresden, Germany; b Department of Internal Medicine III, University Hospital Carl Gustav Carus, Dresden University of Technology, Dresden, Germany; c Department of Basic and Clinical Neuroscience, Institute of Psychiatry, Psychology and Neuroscience, Maurice Wohl Clinical Neuroscience Institute, King’s College London, London, United Kingdom; d MRC Centre for Neurodevelopmental Disorders, King’s College London, London, United Kingdom

**Keywords:** ADMA, Asymmetric dimethylarginine, DDAH, Dimethylarginine dimethylaminohydrolases, Nitric oxide, Psychiatric disorders, Transdiagnostic

## Abstract

The heterogeneous nature of psychiatric disorders complicates their clinical management and the development of novel treatments, imposing a significant burden on both patients and health care systems. To address these challenges, it is essential to continuously identify new targets involved in their pathogenesis. In this narrative review, we propose the dimethylarginine dimethylaminohydrolase (DDAH) proteins, already known for their significant role in cardiovascular disease, as promising novel treatment targets for psychiatric conditions. The DDAH proteins exist in 2 isoforms, DDAH1 and DDAH2, which both regulate nitric oxide homeostasis. DDAH1 metabolizes the nitric oxide synthase inhibitor asymmetric dimethylarginine (ADMA), while DDAH2 acts through ADMA-independent mechanisms. We synthesize current evidence from systemic studies, genetic analyses, postmortem brain samples, and animal models to evaluate the potential roles of DDAH proteins across psychiatric conditions. Most systemic studies have revealed increased peripheral ADMA levels across several psychiatric disorders, including schizophrenia, depression, bipolar disorder, substance use disorders, and attention-deficit/hyperactivity disorder. Alterations in ADMA levels are also observed in transdiagnostic clinical domains such as cognitive deficits, sleep disturbances, white matter hyperintensities, and oxidative stress. These ADMA changes are evident from early stages of illness and respond to current treatments, suggesting diagnostic potential. Genetic and postmortem brain data further link DDAH1 and DDAH2 to psychiatric symptomatology in patient populations. Finally, fundamental studies in model systems provide insights into their role in neural proliferation, differentiation, cell death, and oxidative stress regulation—key processes in the developing and the adult brain. These data support the view that DDAH proteins may play a role in the molecular mechanisms that underlie psychiatric disorders and merit more investigation as potential therapeutic candidates.

## Plain Language Summary

In this review, we highlight the relevance of DDAH proteins, key regulators of nitric oxide balance, for psychiatric research. Evidence from genetic studies, brain tissue, and animal models links DDAH1 and DDAH2 to core psychiatric symptoms, supporting their relevance across disorders and underscoring the need for further investigation.

“I don’t want to be at the mercy of my emotions. I want to use them, to enjoy them, and to dominate them” (Oscar Wilde, *The Picture of Dorian Gray*, 1890); today, this quote still describes the challenge that psychiatric patients face in their everyday lives. Psychiatric disorders are among the leading causes of morbidity (1) and mortality (2). Despite ongoing research, challenges in early diagnosis and accurate treatment persist. Current treatment advances focus on the repurposing of drugs, such as KarXT for schizophrenia (3). While existing modalities can provide clinically meaningful effects (4,5), a lack of therapeutic response, relapse, and adverse effects are common (6,7). Similarly, psychiatric nosology still relies on traditional diagnostic distinctions based on clinical observations (8,9), highlighting the absence of objective biomarkers. Thus, there remains an unmet need for advancements to improve clinical practice.

Genetic underpinnings form the basis for identifying molecular, cellular, and biochemical targets. Twin, family, and genome-wide association studies (GWASs) have shown that psychiatric disorders are heritable and highly polygenic, involving hundreds of risk variants (10,11). Psychiatric disorders and behavioral traits are influenced by a larger number of genetic variations compared to somatic traits, despite similar overall heritability explained by single nucleotide polymorphisms (SNPs) (12, 13, 14) and further shaped by environmental factors (15). Additionally, known genetic risk variants are shared across different psychiatric conditions (16,17) and overlap with traits related to cognition, neuroanatomical, neurological, and immunological phenotypes, as well as cardiovascular and metabolic risk (18). This suggests mechanistic commonalities and explains the high comorbidity that has been observed both among psychiatric disorders and between psychiatric and somatic diseases (16,18). Such pleiotropy underscores the need for a multimodal approach that incorporates genetics, neuroimaging, cognition, circadian rhythms, comorbidities, and physical health, supported by frameworks such as the Hierarchical Taxonomy of Psychopathology and Research Domain Criteria (19,20).

**Figure 1:**
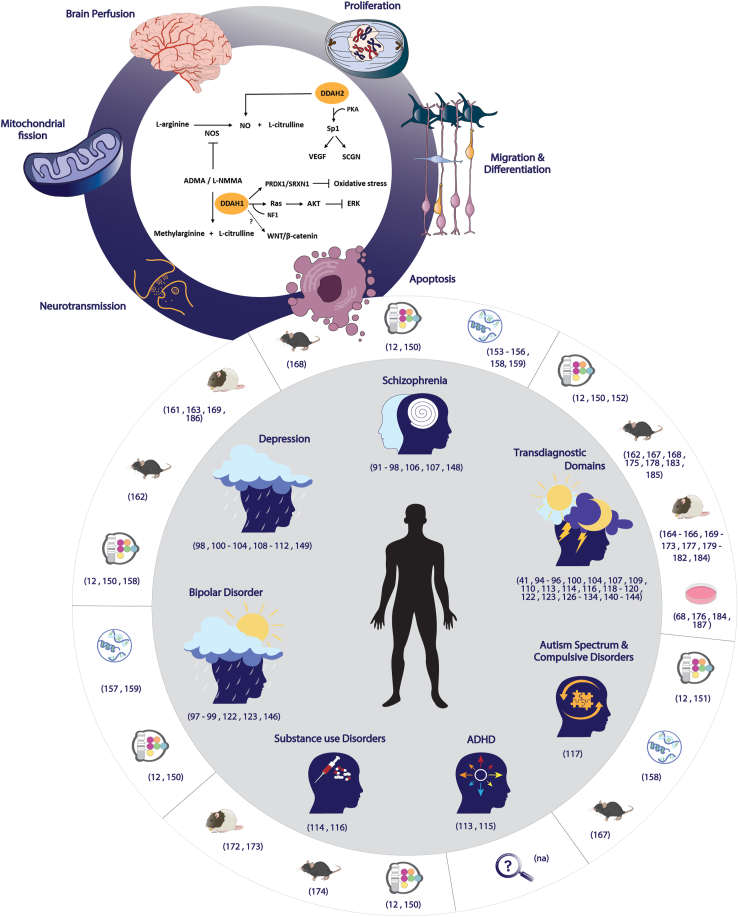
DDAH proteins in psychiatric disorders and relevant transdiagnostic domains. The figure features a small circle (top left) illustrating pathways involving DDAH proteins, including both NO-dependent and alternative targets (inner section), together with an overview of the core functions associated with these targets (outer section). The central circle is divided into 2 parts: The inner section (light gray) highlights the range of psychiatric disorders for which there is systemic clinical evidence implicating DDAH proteins; the outer section (white) presents additional levels of evidence from genetic studies (genome-wide association studies symbol), brain postmortem data (DNA, RNA, protein symbol), as well as in vivo and in vitro preclinical models (rat, mouse, and cell culture symbol) regarding the role of DDAH proteins in psychiatric disorders. ADHD, attention-deficit/hyperactivity disorder; ADMA, asymmetric dimethylarginine; AKT, protein kinase B; ERK, extracellular signal-regulated kinase; L-NMMA, monomethylarginine; NF1, neurofibromin; NO, nitric oxide; NOS, nitric oxide synthase; PKA, protein kinase A; PRDX1, peroxiredoxin 1; SCGN, secretagogin; VEGF, vascular endothelial growth factor.

Given the intricate interplay between polygenicity and pleiotropy, there is an imperative to explore genetic targets and their roles in symptoms, specifically when linked to somatic phenotypes. Dimethylarginine dimethylaminohydrolase (DDAH) proteins have significantly advanced our understanding of cardiovascular disease mechanisms (21, 22, 23, 24, 25) and are valued as diagnostic markers (26) and therapeutic targets (27). Cardiovascular disease is one of the most common comorbidities in psychiatric disorders, with affected individuals exhibiting higher rates of hypertension, diabetes, dyslipidemia, metabolic syndrome, smoking, and physical inactivity. These factors contribute to a cardiovascular mortality rate twice that of the general population. Consistent with these findings, psychiatric patients show an increased incidence of coronary artery disease, cerebrovascular disease, and heart failure, making DDAH proteins candidates for further investigation (28). In support of this idea, DDAH proteins have been linked to various neurological and neurodegenerative disorders, such as Alzheimer’s disease (29, 30, 31, 32), amyotrophic lateral sclerosis (33,34), multiple sclerosis (35), Parkinson’s disease (36,37), Huntington’s disease (38), migraine (39,40), neuropathic pain (41), and epilepsy (42). However, their role in psychiatric disorders remains largely unexplored, with only one meta-analysis reported to date having focused exclusively on schizophrenia (43). Therefore, in this narrative review, we advance the suggestion that DDAH proteins represent promising targets for psychiatric disorders. To ensure comprehensive coverage, we conducted an extensive search across online databases including PubMed, Web of Science, Scopus, Google Scholar, ScienceDirect, Wiley Online Library, and SpringerLink. We used keywords related to the proteins of interest in various combinations with disease-specific terminology. No formal exclusion criteria were applied, and a total of 89 studies, including non-English literature, were evaluated. The literature was independently assessed by 2 raters. We outlined the pathways regulated by DDAH proteins (Figure 1, small circle) and explored clinical evidence from psychiatric cohorts (Figure 1, central circle; Table 1, Table 2, Table 3). While we adhere to the traditional classification systems outlined by the ICD (9) and the DSM (8), we emphasize the relevance of DDAH proteins for broader, transdiagnostic features across diagnostic categories. Finally, we discuss studies in relevant animal models demonstrating endophenotypes associated with psychiatric symptomatology (Figure 1, central circle; Table 4).

**Table 1:** Clinical Studies Involving the DDAH Proteins in Psychiatric Disorders and Relevant Transdiagnostic Domains

Reference	Study Type	Demographics	Sample	Finding	Relevance
Das *et al.*, 1996 (91)	Longitudinal	*N =* 28; female/male = 2/26; age, years = 29.0 ± 1.6	Plasma	ADMA: P (N) < P > HC NO: P^a^ (N) > P < HC	Schizophrenia
Selley, 2004 (100)	Cross-sectional	*N =* 50; female/male = 25/25; age, years = 46.1 ± 12.4	Plasma	ADMA: P > HC NO: P < HC	Depression, oxidative stress
Ohike *et al.*, 2005 (147)	Longitudinal	*N =* 10; female/male = 0/10; age, years = 53.3 ± 10.5	Plasma	ADMA: P (PAP) < P NO: P (PAP) = P	Sleep disorder
Gozal *et al.*, 2007 (133)	Longitudinal	*N =* 34; female/male = 13/21; age, years = 6.9 ± 0.6	Plasma	ADMA: P = HC NO: P = HC	Sleep disorder
Khan *et al.*, 2007 (142)	Cross-sectional	*N =* 85; female/male = 34/51; age, years = 65.3 ± 10.4	Plasma	ADMA: P > HC	WMH
Ozkan *et al.*, 2008 (132)	Cross-sectional	*N =* 49; female/male = 13/36; age, years = 46.8 ± 13.2	Serum, plasma	ADMA: P = HC (high trend) NO: P < HC	Sleep disorder, oxidative stress
Barceló *et al.*, 2009 (126)	Cross-sectional	*N =* 64; female/male = 0/64; age, years = 46.8 ± 9.13	Plasma	ADMA: P > HC	Sleep disorder
Pikula *et al.*, 2009 (143)	Longitudinal	*N =* 2013; female/male = 1067/946; age, years = 58.0 ± 9.5	Plasma	Positive correlation between ADMA and silent brain infarcts (community-based)	WMH
Piletz *et al.*, 2009 (108)	Longitudinal	*N =* 30; female/male = 25/5; age, years = 39.5 ± 2.1	Plasma	ADMA: P (V) = P = HC NO: P (V) = P = HC	Depression
Celik *et al.*, 2011 (92)	Cross-sectional	*N =* 79; female/male = 0/79; age, years = 23.9 ± 5.6	Plasma	ADMA: P > HC; FES < MES	Schizophrenia
Aykut *et al.*, 2012 (99)	Cross-sectional	*N =* 60; female/male = 30/30; age, years = 37.6 ± 13.6	Plasma	ADMA: P > HC NO: P < HC	Bipolar disorder
Di Gennaro *et al.*, 2012 (114)	Longitudinal	*N =* 64; female/male = 10/54; age, years = 44.8 ± 9.4	Plasma	ADMA: P (detox) > P > HC	SUD, oxidative stress
Frieling *et al.*, 2012 (116)	Longitudinal	*N =* 74; female/male = 0/74; age, years = 45.8 ± 8.3	Plasma	ADMA: P (detox) > P < HC	SUD, oxidative stress
McEvoy *et al.*, 2013 (101)	Longitudinal	*N =* 347; female/male = 176/171; age, years = 64.0 ± 7.4	Serum	ADMA: P > HC	Depression
Miralbell *et al.*, 2013 (120)	Cross-sectional	*N =* 747; female/male = 255/492; age, years = 66.1 ± 7.6	Serum	Negative correlation between ADMA and verbal memory (HC)	Cognitive function
Baranyi *et al.*, 2014 (102)	Longitudinal	*N =* 41; female/male = 17/24; age, years = 43.7 ± 14.1	Plasma	ADMA: P (IFN-α) > hepatitis C	Depression
Canpolat *et al.*, 2014 (109)	Cross-sectional	*N =* 85; female/male = 58/27; age, years = 26.3 ± 4.9	Serum	ADMA: P = HC NO: P = HC	Depression
McEvoy *et al.*, 2014 (119)	Cross-sectional	*N =* 483; female/male = 261/222; age, years = 64.0 ± 7.4	Serum	Positive correlation between ADMA and subjective memory impairment in aging cohort (HC)	Cognitive function
Raw *et al.*, 2014 (110)	Longitudinal	*N =* 63; female/male = 63/0; age, years = 22.5 ± 4.4	Plasma	ADMA: P < HC	Depression, oxidative stress
Zincir *et al.*, 2014 (93)	Longitudinal	*N =* 79; female/male = not available; age, years = not available	Plasma	ADMA: P (A) < P > HC	Schizophrenia
Aribas *et al.*, 2015 (128)	Cross-sectional	*N =* 78; female/male = 48/30; age, years = 48.8 ± 9.4	Serum	ADMA: poor sleep > good sleep	Sleep disorder
Baranyi *et al.*, 2015 (103)	Longitudinal	*N =* 119; female/male = 40/79; age, years = 49.7 ± 9.6	Plasma	ADMA: P (H) = P > HC	Depression
Gao *et al.*, 2015 (141)	Cross-sectional	*N =* 417; female/male = 205/212; age, years = 76.0 ± 11.0	Serum	ADMA: P > HC	WMH
In *et al.*, 2015 (127)	Cross-sectional	*N =* 60; female/male = 17/43; age, years = 53.4 ± 11.5	Serum	ADMA: P > HC	Sleep disorder
Jorgensen *et al.*, 2015 (107)	Cross-sectional	*N =* 80; female/male = 40/40; age, years = 33.0 ± 10.7	Plasma	ADMA: P (A) = HC	Schizophrenia, oxidative stress
Kicinski *et al.*, 2015 (134)	Cross-sectional	*N =* 59; female/male = 16/43; age, years = 53.6 ± 11.2	Serum	ADMA: P = HC	Sleep disorder
Mommersteeg *et al.*, 2015 (112)	Longitudinal	*N =* 104; female/male = 29/75; age, years = 65.8 ± 8.3	Serum	ADMA: P (baseline) = P (12 mo)	Depression
Nonaka-Hashida, 2016 (148)	Cross-sectional	*N =* 164; female/male = 69/95; age, years = 50.3 ± 13.8	Plasma	ADMA: P (A) > HC	Schizophrenia
Telo and Gurok, 2016 (95)	Cross-sectional	*N =* 120; female/male *=* 58/62; age, years = 42.6 ± 11.8	Serum, plasma	ADMA: P (N^b^) > HC	Schizophrenia, oxidative stress
Yang *et al.*, 2016 (94)	Cross-sectional	*N* = 92; female/male = 46/46; age, years = 30.2 ± 8.5	Plasma	ADMA: P > HC; FES < MES	Schizophrenia, cognitive function
Yilmaz *et al.*, 2016 (117)	Cross-sectional	*N =* 60; female/male *=* 35/25; age, years = 33.3 ± 10.4	Serum	ADMA: P < HC NO: P = HC (high trend)	OCD
Erdélyi-Bótor *et al.*, 2017 (40)	Cross-sectional	*N =* 89; female/male *=* 71/18; age, years = 36.7 ± 10.8	Serum	ADMA: P (WMH) > HC	WMH
Guan *et al.*, 2017 (140)	Cross-sectional	*N =* 148; female/male *=* 70/78; age, years = 73.97 ± 9.12	Serum	ADMA: P (WMH) > HC	WMH
Oglodek, 2017 (104)	Cross-sectional	*N =* 460; female/male *=* 120/120; age, years = 45.2 ± 4.5	Serum	ADMA: P (severe + PTSD) > P (severe) > P (moderate) > P (mild) > HC	Depression, PTSD, oxidative stress
Tiryaki *et al.*, 2017 (146)	Longitudinal	*N =* 17; female/male *=* 9/8; age, years = 36.2 ± 14.2	Serum	ADMA: P (L) < P NO: P (L) > P	Bipolar disorder
Ali-Sisto *et al.*, 2018 (149)	Longitudinal	*N =* 352; female/male = 185/167; age, years = 39.4 ± 11.9	Serum	ADMA: P (T) = HC P (Trem) = P (Tnon-rem)	Depression
Bani-Fatemi *et al.*, 2018 (156)	Cross-sectional (EWAS)	*N =* 123; female/male = 37/86; age, years = 44.7 ± 12.3	White blood cells	Increased methylation of *DDAH2*’s regulatory regions	Schizophrenia, suicide attempts
Gürsoy *et al.*, 2018 (122)	Cross-sectional	*N =* 90; female/male = 38/52; age, years = 38.3 ± 12.0	Serum	ADMA: P (T^b^) < HC NO: P (T^b^) = HC	Bipolar disorder
Obayashi *et al.*, 2018 (129)	Cross-sectional	*N =* 1115; female/male = 588/527; age, years = 71.9 ± 7.1	Serum	Worse sleep parameters in high ADMA group (only females)	Sleep disorder
Arlouskaya *et al.*, 2019 (130)	Cross-sectional	*N =* 518; female/male = 384/134; age, years = 43.8 ± 11.3	Serum	ADMA: P > HC	Sleep disorder
Janes *et al.*, 2019 (144)	Cross-sectional	*N =* 70; female/male = 58/12; age, years = 51.1 ± 9.3	Plasma	ADMA: P (WMH) > HC	WMH
Safaei *et al.*, 2019 (106)	Cross-sectional	*N =* 77; female/male = 23/54; age, years = 46.1 ± 12.5	Serum	ADMA: P (A) > P = HC P (Cloz.) > P (Risp.) = P = HC	Schizophrenia
Yu *et al.*, 2019 (96)	Longitudinal	*N =* 59; female/male = 29/30; age, years = 31.2 ± 8.2	Plasma	ADMA: P (A) < P > HC	Schizophrenia, cognitive function
Jansen *et al.* 2020 (115)	Cross-sectional	*N =* 85; female/male = 24/61; age, years = 9.1 ± 2.4	Plasma	ADMA: P (MPH) = P < HC NO: P (MPH) > P > HC	ADHD
Malden *et al.*, 2020 (118)	Longitudinal	*N =* 93; female/male = 42/51; age, years = 63.3 ± 0.6	Plasma	Negative correlation between ADMA and cognitive function (HC)	Cognitive function
Ozden *et al.*, 2020 (111)	Cross-sectional	*N =* 104; female/male = 69/35; age, years = 41.1 ± 12.2	Plasma	ADMA: P < HC	Depression
Ustundag *et al.*, 2020 (97)	Cross-sectional	*N =* 90; female/male = 50/40; age, years = 37.6 ± 9.3	Serum	ADMA: P (T^b^) > HC NO: P < HC (schizophrenia) NO: P (T^b^) = HC (bipolar disorder)	Schizophrenia, bipolar disorder
Braun *et al.*, 2021 (98)	Cross-sectional	*N =* 154; female/male = 81/73; age, years = 46.0 ± 18.8	Serum	ADMA: P (T) > HC	Schizophrenia, bipolar disorder, depression
Tozoglu *et al.*, 2021 (123)	Cross-sectional	*N =* 72; female/male = 35/37; age, years = 36.1 ± 9.6	Serum	ADMA: P (T^b^) < HC NO: P (T^b^) = HC (lower trend)	Bipolar disorder, oxidative stress
Doneray *et al.*, 2022 (113)	Longitudinal	*N =* 60; female/male = 26/34; age, years = 10.0 ± 1.6	Serum	ADMA: P (MPH) < P > HC NO: P (MPH) = P < HC	ADHD, oxidative stress
Safci *et al.*, 2022 (131)	Cross-sectional	*N =* 330; female/male = 23/307; age, years = 44.2 ± 14.2	Serum	ADMA: P > HC	Sleep disorder

Demographics of study participants include overall sample size (*N*), sex, and age presented as mean ± SD for all included patients. When not available from the original references, the mean and SD were calculated from patient subgroups using weighted means and pooled SDs. Patients under treatment are denoted by P with the treatment agent specified in parentheses.

A, atypical antipsychotics; ADHD, attention-deficit/hyperactivity disorder; ADMA, asymmetric dimethylarginine; Cloz., clozapine; detox, detoxification treatment; EWAS, epigenome-wide association study; FES, first-episode patient; H, hospitalization; HC, healthy control participant; IFN-α, interferon alpha; L, lithium; MES, multiple-episode patient; MPH, methylphenidate; N, neuroleptic; NO, nitric oxide; OCD, obsessive-compulsive disorder; P, patient; PAP, positive airway pressure; PTSD, posttraumatic stress disorder; Risp., risperidone; SUD, substance use disorder; T, unspecified or various mixed treatment; Tnon-rem, non-remitted patient under unspecified or various treatments; Trem, remitted patient under unspecified or various treatments; V, venlafaxine; WMH, white matter hyperintensity.

a Only 3 patients received treatment.

b Patients on various treatment regimens.

**Table 2:** GWASs Involving *DDAH* Gene Variants in Psychiatric Disorders and Relevant Transdiagnostic Domains

PMID	Year	Gene	Sample Size	*p* Value	Relevance
17728769	2007	*DDAH2*	3554	.043	Depression
17903297	2007	*DDAH1*	327	.057	Hippocampal volume
			705	.040	WMH volume
			694	<.022	Cognitive functions
17903308	2007	*DDAH1*	736	.011	Sleep duration
20418890	2010	*DDAH2*	41,278	.008	Smoking behavior
21926974	2011	*DDAH1*	21,856	.006	Schizophrenia
		*DDAH2*		<.001	
22952603	2012	*DDAH1*	381	<.001	Amphetamine response
22978509	2012	*DDAH1*	1161	.037	Alcohol dependence
23089632	2013	*DDAH1*	2322	<.001	Alcohol dependence
23453885	2013	*DDAH2*	61,220	.001	Shared genetic risk across major psychiatric disorders
23974872	2013	*DDAH1*	32,143	.003	Schizophrenia
		*DDAH2*		<.001	
24280982	2014	*DDAH1*	16,381	.024	Schizophrenia vs. bipolar disorder (genetic distinction)
		*DDAH2*		<.001	
		*DDAH2*	39,202	<.001	Schizophrenia/bipolar disorder (shared genetic risk)
24369049	2014	*DDAH1*	294	.005	Lithium response
25056061	2014	*DDAH1*	82,315	.024	Schizophrenia
		*DDAH2*		<.001	
27046643	2016	*DDAH1*	111,483	.049	Processing speed/reaction time
27089181	2016	*DDAH2*	170,911	.038	Neuroticism
27656889	2016	*DDAH1*	1655	.036	Attention/executive function
27694991	2016	*DDAH2*	26,577	.015	Intracranial volume
28540026	2017	*DDAH1*	15,954	.015	Autism spectrum disorder
28641744	2017	*DDAH1*	9896	<.001	Autism spectrum disorder, obsessive-compulsive disorder
29255261	2018	*DDAH2*	329,821	.001	Neuroticism
29483656	2018	*DDAH1*	105,318	.040	Schizophrenia
		*DDAH2*		<.001	
29500382	2018	*DDAH1*	265,139	.005	Feelings of guilt
		*DDAH2*		.002	
		*DDAH2*	380,506	<.001	Neuroticism, affective traits/emotional sensitivity
			262,321–267,050	<.001–.026	
29662059	2018	*DDAH2*	322,580	.005	Depression
29700475	2018	*DDAH2*	173,005	.002	Depression
29844566	2018	*DDAH2*	168,033	.035	Logical analysis/problem solving
29906448	2018	*DDAH1*	87,491	.008	Schizophrenia
		*DDAH2*		<.001	
		*DDAH1*	38,855	<.001	Schizophrenia vs. bipolar disorder (genetic distinction)
		*DDAH2*		<.001	
		*DDAH1*	74,194	.025	Bipolar disorder
		*DDAH2*	107,620	<.001	Schizophrenia/bipolar disorder (shared genetic risk)
29942085	2018	*DDAH2*	390,278	<.001	Neuroticism
			357,957	.020	Depression,
			348,219	<.001	Difficulty controlling worry (anxiety)
29942086	2018	*DDAH1*	269,867	.007	General cognitive ability (intelligence)
30482948	2018	*DDAH2*	52,848	.008	Alcohol dependence
30531941	2018	*DDAH1*	91,105	<.001	Movement inactivity during sleep
30643251	2019	*DDAH2*	632,802	.005	Smoking behavior
30696823	2019	*DDAH2*	449,732	.02	Chronotype
30804565	2019	*DDAH1*	345,552	.048	Chronotype (morning)
		*DDAH2*		.005	
30804566	2019	*DDAH1*	237,627	.030	Sleep disorder (insomnia)
		*DDAH2*		.013	
30846698	2019	*DDAH2*	446,118	.008	Sleep duration
30867560	2020	*DDAH2*	270,059	.024	Neuroticism
				<.001	Difficulty controlling worry (anxiety)
30952852	2019	*DDAH1*	84,810	.006	Sleep efficiency, sleep episodes
		*DDAH2*	84,441	<.001	Sleep duration
30995994	2019	*DDAH2*	441	.014	Language processing (memory, attention)
31427789	2019	*DDAH1*	386,082	.004	Alcohol consumption
		*DDAH2*		.032	
		*DDAH1*	65,776	.009	Depression
		*DDAH2*	71,568	.018	
		*DDAH1*	376,361	.006	Feelings of guilt
		*DDAH2*		.050	
		*DDAH1*	345,148	.041	Chronotype
		*DDAH2*		.009	
		*DDAH1*	126,300	.020	Difficulty controlling worry (anxiety)
		*DDAH2*	376,411	<.001	
		*DDAH1*	68,065	.040	Cognitive ability
		*DDAH2*	355,594	.001	Smoking status
		*DDAH2*	128,912	.043	Prospective memory/memory retrieval
		*DDAH2*	95,708	.002	Visual memory/working memory
31676860	2019	*DDAH1*	19,629–21,821	<.001–.050	Volume of multiple brain regions
		*DDAH2*			
32665545	2020	*DDAH1*	26,502	<.001	Cortical surface area
33433489	2021	*DDAH2*	92	<.001	WMH
33821002	2021	*DDAH1*	19,670	<.001	Brain shape
34560273	2021	*DDAH1*	35,657	<.001	Cortical surface area, cortical thickness
34910505	2021	*DDAH1*	33,748	<.001	Vertexwise sulcal depth, vertexwise cortical surface area
35164939	2022	*DDAH1*	33,735	<.001	Brain morphology
36893272	2023	*DDAH2*	32,488	<.001	Brain morphology
37089073	2023	*DDAH1*	2995	<.001	Cognitive performance

Preprints were not included. The information has been extracted from (12,150, 151, 152). In cases where traits were grouped, we provided the corresponding range of sample sizes and associated *p* values for the pooled traits.

GWAS, genome-wide association study; PMID, PubMed identifier; WMH, white matter hyperintensity.

**Table 3:** Brain Postmortem Studies Involving *DDAH* Genes in Psychiatric Disorders and Relevant Transdiagnostic Domains

Reference	Study Type	Demographics	Sample	Finding	Relevance
Clark *et al.*, 2006 (153)	Proteome	*N =* 20; female/male = 4/16; age, years = 48.0 ± 10.7	ACC	*DDAH1*: P < HC	Schizophrenia, oxidative stress
Narayan *et al.*, 2008 (154)	Transcriptome	*N =* 55; female/male = 11/44; age, years = 44.2 ± 2.91	PFC	*DDAH1*: P < HC (only early stage)	Schizophrenia, gene expression, development, cell signaling, carbohydrate metabolism
Wang *et al.*, 2016 (157)	Transcriptome meta-analysis	*N =* 207; female/male = 80/127; age, years = 44.4 ± 10.5	PFC	*DDAH2*: P > HC	Bipolar disorder, apoptosis
Wu *et al.*, 2021 (155)	Epigenome/transcriptome meta-analysis	*N =* 1211; female/male = 382/829; age, years = 50.9 ± 17.9	FC	Reduced methylation near the TSS of *DDAH2, DDAH2*: P > HC	Schizophrenia, apoptosis, cell signaling, metabolic functions
Kozlova *et al.*, 2022 (159)	Transcriptome meta-analysis	*N =* 34; female/male = 9/25; age, years = 46.2 ± 9.7	DLPFC	Functional shifts in *DDAH1* and *DDAH2* coexpression modules	Schizophrenia, bipolar disorder
Pineda-Cirera *et al.*, 2022 (158)	Epigenome/transcriptome meta-analysis	*N =* 303; female/male = 103/200; age, years = 45.0 ± 17.5	Cb, FC	Reduced methylation *DDAH2*: P > HC	Schizophrenia, depression, compulsive behaviors

Demographic characteristics of study participants include overall sample size (*N*), sex, and age presented as mean ± SD for all included patients. When not available from the original references, the mean and SD were calculated from patient subgroups using weighted means and pooled SDs.

ACC, anterior cingulate cortex; Cb, cerebellum; DLPFC, dorsolateral prefrontal cortex; FC, frontal cortex; HC, healthy control participant; P, patient; TSS, transcription start site.

**Table 4:** In Vivo and In Vitro Preclinical Studies Involving the DDAH Proteins in Psychiatric Disorder–Relevant Phenotypes

Reference	Model	Sample	Finding	Relevance
Khawaja *et al.*, 2004 (186)	Healthy rat	Hip, proteome	DDAH1: C (MARI) > C	Descriptive, chronic antidepressant treatment effect
Tomikawa *et al.*, 2006 (68)	Healthy mouse embryos	troph. cells	*Ddah2*→hypermethylated in troph. stem cells→hypomethylated in differentiated troph. cells	Descriptive, development, differentiation
Li *et al.*, 2008 (172)	METH-treated rat	FC, Str, Hip, proteome	DDAH1: M > C (FC, Str)	Descriptive, SUD, oxidative stress
Bäckdahl *et al.*, 2009 (187)	Induction of differentiation	E14 stem cells, mouse embryos	*Ddah2*→hypomethylated in stem cells →hypermethylated in differentiated neuronal stem cells and neurons	Descriptive, development, differentiation
Marais *et al.*, 2009 (161)	Maternal separation rat	vHip, proteome	DDAH1: M (MARI) > M < C	Descriptive, depressive-like
Gunduz *et al.*, 2010 (174)	Morphine-treated mouse	Plasma	ADMA: M = C	Descriptive, SUD
Huang *et al.*, 2010 (180)	Bile duct ligation rat	Plasma, cortex	ADMA (plasma): M > C DDAH1 (cortex): M = C	Descriptive, learning and memory deficits
Amrouni *et al.*, 2011 (170)	*T. b. brucei–*infected rat	Dienceph., plasma	DDAH: M > C (activity, dienceph.) DDAH1: M = C (dienceph.) DDAH2: M > C (dienceph.) ADMA: M > C (plasma)	Descriptive, sleep disorder, inflammation
Whittle *et al.*, 2011 (162)	Mg-restricted mouse	Hyp, Amg, proteome	DDAH1: M (MARI) > M < C	Descriptive, depressive-like, oxidative stress
Wang *et al.*, 2012 (176)	NGF-promoted differentiation	PC12 cells	During NGF-promoted differentiation: DDAH1→increase, neurite formation/DDAH2→decrease	Descriptive, development, differentiation
Cui *et al.*, 2013 (164)	Monosodium L-glutamate-treated rat	Hip	DDAH1: M (T) > M < C	Descriptive, anxiety-like, oxidative stress
Hirotsu *et al.*, 2013 (171)	Total and paradoxical sleep deprivation, rat	FC	*Ddah1*: M > C (total sleep, mRNA) *Nos3* (eNOS): M > C (total sleep, mRNA)	Descriptive, sleep disorder, oxidative stress
Zhang *et al.*, 2013 (173)	METH-treated rat, PC12 cells	FC, Str, Hip	DDAH1: M (L-257) < M > C (FC, Str) ADMA: M (L-257) > M < C (all) NOS/NO: M (L-257) < M > C (all)	Descriptive, SUD, oxidative stress
Kielstein *et al.*, 2015 (163)	Chronic subcutaneous ADMA infusion	Plasma	ADMA: M > C	Descriptive, depressive-like
Hsu *et al.*, 2018 (181)	Bile duct ligation rat	Plasma, PFC, dHip	ADMA: M (melatonin) < M > C	Descriptive, learning and memory deficits
Xiao *et al.*, 2018 (165)	Sleep-deprived rat	Dienceph.	DDAH1/2: M (T) > M < C ADMA: M (T) < M > C	Descriptive, sleep disorder, oxidative stress
Sheen *et al.*, 2019 (179)	Healthy rat, ADMA infusion	Plasma, dHip	ADMA: M (melatonin) < M > C	Descriptive, learning and memory deficits, oxidative stress
Xiao *et al.*, 2019 (166)	Sleep-deprived rat	Dienceph.	DDAH1/2: M (T) > M < C ADMA: M (T) < M > C nNOS/NO: M (T) > M < C	Descriptive, sleep disorder, oxidative stress
Choi *et al.*, 2020 (178)	Cerebrovascular β-amyloidosis mouse model, ADMA infusion	Serum, brain	ADMA: M > C N-Tyr: M > C	Descriptive, learning, and memory deficits, BBB, nitrosative stress, inflammation
Cortelazzo *et al.*, 2020 (167)	*Mecp2* null mouse model	Brain, proteome	DDAH1: M (gene rescue) > M < C	Descriptive, autism-like behavior, oxidative stress
Cieślik *et al.*, 2021 (168)	MK-801/scopolamine mouse	PFC, Hip	DDAH1: M > C	Descriptive, learning and memory deficits, schizophrenia-like (MK-801)
Ploska *et al.*, 2021 (169)	Olfactory bulbectomized rat	PFC, Hip, Str	DDAH1: M (CDPPB) < M > C (PFC) DDAH1: M (CDPPB) < M = C (Hip, Str)	Descriptive, learning and memory deficits, depressive-like
Singh *et al.*, 2021 (183)	EAE mouse model, ADMA infusion	Serum, brain, spinal cord	ADMA (serum): M > C (in EAE)	Descriptive, BBB, inflammation
Zhao *et al.*, 2021 (182)	*Ddah1* KO rat, induction of MCAO	Brain, plasma	ADMA: M > C NO: M < C	Genetic, BBB
Hsu *et al.*, 2023 (177)	Healthy rat, ADMA infusion	dHip, plasma	ADMA: M (resveratrol) < M > C	Descriptive, learning, and memory deficits, BBB, inflammation
Kozlova *et al.*, 2023 (185)	*Ddah1* KO mouse	Brain	DDAH1 depletion→reduced amphetamine sensitivity	Genetic, dopamine system
Gao *et al.*, 2024 (175)	*Ddah1* KO/TG mouse, induction of MCAO	Brain	DDAH1 regulates genes for acetylcholine synthesis and transport through HIF-1α	Genetic, proliferation, neural differentiation
Zhao *et al.*, 2024 (184)	*Ddah1* KO rat, rotenone injections	Brain, PC12 cells	DDAH1 depletion→rotenone -induced dopaminergic neurodegeneration	Genetic, mitochondrial dysfunction

Animal models are indicated as M, treatment models are indicated as M with the treatment specified in parentheses, and controls are indicated as C. CDPPB is a positive allosteric modulator of the mGlu_5_ receptor, and L-257 is a DDAH1 inhibitor *N*(ω)-(2-methoxyethyl)-arginine. The second methylated L-arginine derivative, monomethylarginine, although potentially relevant, has not been reported here because it has not been evaluated in in vivo and in vitro preclinical studies.

ADMA, asymmetric dimethylarginine; Amg, amygdala; BBB, blood-brain barrier; C, control; dHip, dorsal hippocampus; dienceph., diencephalon; EAE, experimental autoimmune encephalomyelitis; eNOS, endothelial nitric oxide synthase (encoded by *Nos3* gene); FC, frontal cortex; Hyp, hypothalamus; KO, knockout; M, animal model; MARI, monoamine reuptake inhibitor; MCAO, middle cerebral artery occlusion; METH, methamphetamine; Mg, magnesium; mRNA, messenger RNA; NGF, nerve growth factor; NO, nitric oxide; NOS, nitric oxide synthase; nNOS, neuronal NOS; N-Tyr, nitrotyrosine; PFC, prefrontal cortex; Str, striatum; SUD, substance use disorder; T, treatment; TG, transgenic; troph., trophoblast; vHip, ventral hippocampus.

## DDAH Proteins Regulate Major Signal Transduction Pathways Relevant to Psychiatric Disorders

The *DDAH* genes, located on chromosomes 1p22 and 6p21.3 in the human genome, encode 2 proteins, DDAH1 and DDAH2, respectively (44). These isoforms are both involved in maintaining nitric oxide (NO) homeostasis with no alternatively spliced forms. NO is a gaseous molecule synthesized from the oxidation of L-arginine to L-citrulline in a 2-step redox reaction catalyzed by NO synthase (NOS) isoforms: neuronal NOS (nNOS), endothelial NOS (eNOS), or inducible NOS (iNOS). This reaction can be inhibited by methylated L-arginine analogs, such as asymmetric dimethylarginine (ADMA) and monomethylarginine (L-NMMA), which compete with L-arginine for the binding site on NOS enzymes, thereby reducing NO synthesis. In turn, DDAH1 metabolizes these primary endogenous NOS inhibitors, restoring balance and serving as a positive regulator of NO (45,46). Recent work shows that DDAH2 does not hydrolyze ADMA but may influence NO levels through an ADMA-independent pathway that remains poorly understood (47) (Figure 1, small circle, inner section). DDAH2 regulation of NO is evident in its role in NO-mediated mitochondrial fission (48). A recent review summarizes the biochemical data suggesting that DDAH2 may act as a serine protease and nonenzymatic regulator (49).

Beyond their role in NO regulation, both DDAH proteins have alternative molecular targets. DDAH1 directly interacts with the guanine triphosphatase (GTPase) activator protein neurofibromin, which maintains Ras in its active form (50). Ras activation subsequently leads to the phosphorylation of downstream effectors, such as protein kinase B (AKT), with studies reporting a positive correlation between DDAH1 and phosphorylated AKT levels (51, 52, 53). Additionally, DDAH1 correlates with components of the ERK (51,54) and WNT/β-catenin (55) pathways, likely mediated through AKT. However, the involvement of the primary NO pathway cannot be definitively excluded (56). Moreover, DDAH1 recruits enzymes for redox regulation, specifically peroxiredoxin 1 and sulfiredoxin 1, to maintain its expression and activity during oxidative stress and preserve cellular redox homeostasis (57). On the other hand, DDAH2 promotes the expression of vascular endothelial growth factor (VEGF) (58, 59, 60) and secretagogin (61), primarily through interactions with protein kinase A and the transcription factor Sp1 (59,61). As the *NOS* gene promoter contains an Sp1-dependent enhancer (62,63), Sp1 could also serve as a mechanism for regulating NO levels independently of ADMA (Figure 1, small circle, inner section).

Both DDAH isoforms are expressed in the central nervous system in humans and rodents in a region- and cell-specific manner. DDAH1 messenger RNA (mRNA) and protein expression increases from pre- to postnatal stages exhibiting a broad pattern, specifically in neurons, astrocytes, and endothelial cells of blood vessels (44,64, 65, 66, 67). In contrast, DDAH2 shows high expression in the fetal brain and a reduced, limited expression in adults (44,64,66,68). This pattern suggests that DDAH1 is the principal isoform in the postnatal brain, while DDAH2 may play a more significant role in developmental processes. Consistent with this, the molecular targets regulated by DDAH proteins can influence both developmental processes and functions throughout adulthood (Figure 1, small circle, outer section). Specifically, NO modulates neurogenesis, neural and glial differentiation, and a spectrum of homeostatic functions including food intake, sleep regulation, learning, memory, hormone release, synaptic transmission, neurosecretion, brain perfusion, and thermal regulation. These effects arise from NO’s function as a neurotransmitter, its involvement in multiple downstream signaling pathways, and its systemic role as a primary vasodilator. Dysregulated NO levels contribute to neuroinflammation and nitrosative stress, which can lead to developmental abnormalities and cellular damage throughout life (69, 70, 71, 72, 73). Additionally, all alternative DDAH targets act as central signaling hubs engaging in pathways essential for proliferation, migration, differentiation, and apoptosis (74, 75, 76, 77, 78). A substantial body of literature underscores the involvement of the various DDAH downstream targets in psychiatric conditions and associated phenotypes, which has been observed in both patients and animal models (79, 80, 81, 82, 83, 84, 85, 86, 87, 88, 89, 90). While it is informative, this offers only circumstantial evidence for the role of DDAH proteins in psychiatric etiology.

## Building the Case for DDAH Proteins and Their Role in Psychiatric Disorders From Clinical Studies

### From Systemic Evidence to Brain Function

Most data originate from peripheral blood ADMA measurements in diverse cohorts of psychiatric patients (Figure 1, central circle, inner section; Table 1). Das *et al.* first initiated investigations into the DDAH1/ADMA axis within the field of psychiatric disorders. Their work provided a link between manifestation of schizophrenia symptoms and the concurrent increase in plasma ADMA alongside a reduction in NO levels (91). Subsequent studies of schizophrenia (92, 93, 94, 95, 96, 97, 98), bipolar disorder (97, 98, 99), and depression (98,100, 101, 102, 103, 104) consistently supported this discovery. Even after adjusting for sex, age, and metabolic status, ADMA differences remained significant between patients and healthy control participants (94,96,98). Similar alterations in ADMA and NO levels among different diagnostic groups support the notion of common underlying neurobiological mechanisms (97,98). Comparable systemic ADMA changes have also been observed in posttraumatic stress disorder, with the presence of comorbid depression exacerbating this phenotype (104). A population-based study monitoring older adults over 6 years identified ADMA as a significant predictor of future depressive episodes, reinforcing its relevance (101). Sex differences, while of importance (105), have been generally absent in ADMA levels except for one study that noted higher levels in females with depression and posttraumatic stress disorder (104).

In contrast, some studies have reported no changes in ADMA levels between patients and control participants in schizophrenia (106,107) and depression (108,109). Moreover, in depression, a reversed pattern of ADMA directionality has been noted (110,111). However, it is important to consider that the depression studies reviewed here include individuals with comorbid conditions such as heart failure (112), pregnancy-associated depression (110), or depression induced by treatments such as interferon alpha for hepatitis C infection (102). Insufficient research in the areas of attention-deficit/hyperactivity disorder (ADHD), substance use disorders, and obsessive-compulsive disorder prevents drawing definitive conclusions. Specifically, studies on ADHD and alcohol dependence have yielded conflicting results, with some studies suggesting increases (113,114) and others indicating decreases (115,116), while the sole study on obsessive-compulsive disorder demonstrated reduced levels of ADMA (117).

ADMA levels are linked to the diagnosis of the disorder, despite variations in subtypes and phases. For example, patients with paranoid and disorganized schizophrenia (92), as well as schizoaffective disorder (98), all exhibit similarly elevated ADMA levels. Also, data from individuals with bipolar disorder experiencing manic, depressive, and mixed-episode phases indicated equally elevated ADMA concentrations (98). However, patients with recurrent episodes in both schizophrenia (92,94) and depression (98) have shown a more pronounced elevation of ADMA compared with first-episode patients. Despite these findings, disease severity, as estimated by standard clinical scales, did not correlate with ADMA levels in schizophrenia (92, 93, 94, 95, 96), bipolar disorder (97), ADHD (113), and alcohol dependence (116), but it did correlate in depression (101,102,104,111,112). In particular, both positive (101,102,104,112) and negative (111) correlations with ADMA have been reported, reflecting the previously mentioned greater variability of ADMA levels in depression. Additionally, ADMA showed a negative correlation with anxiety and stress severity in patients with depression (111). Standard clinical scales assess observable behaviors and subjective experiences without incorporating more detailed phenotypes. Therefore, the correlations observed in depression may suggest direct involvement of ADMA-modulated pathways in the disorder’s symptomatology, which is sensitive to detection using the standard scales. In contrast, the relationship between ADMA and symptom severity in the other disorders could manifest in broader pathophysiological domains of transdiagnostic value, which may include behavioral impairments in tailored paradigms as well as changes on the neural circuit and molecular level.

Cognitive function assessed through behavioral paradigms is more intricately connected to ADMA. In the general population, a negative correlation between ADMA levels and cognitive performance has been found (118, 119, 120). In schizophrenia, ADMA shows negative correlations with attention, working memory, and executive function, emerging as an independent contributor to deficits in these functional domains (94,96). There was also a trend toward a negative correlation between ADMA and processing speed, while no association was found between ADMA and verbal learning or visual memory (94,96). Alongside the elevated ADMA levels, cognitive dysfunctions were more pronounced in patients with multiple-episode schizophrenia than in patients with drug-naïve first-episode schizophrenia, indicating a worsening of cognitive performance in conjunction with increased ADMA levels (94). These correlations between ADMA and cognitive functions emphasize its potential as an important target, particularly given that addressing cognitive deficits remains a significant unmet need in schizophrenia (121). Conversely, in bipolar disorder, ADMA demonstrates positive correlations with cognitive functions, such as verbal learning and memory, cognitive flexibility, executive function, and attentional control, while the relationship between ADMA and processing speed remained contradictory (122,123). These findings are specific to the euthymic phase, where patients continue to experience cognitive deficits, suggesting that not only high but also low levels of ADMA may be associated with poorer cognitive performance. Cognitive data for the active phases of the disorder are currently unavailable (122,123). Research involving patients with depression has linked lower levels of ADMA to increased impulsivity and reduced attention, while elevated levels of NO were also associated with poorer cognitive performance, particularly in tasks that require attention and inhibition (109). While these behavioral domains are relevant, similar correlations were not confirmed in patients with ADHD (113).

Cognitive health is closely linked to adequate sleep and well-regulated circadian rhythms (124,125), making disturbances in these processes core features across psychiatric disorders. Elevated plasma ADMA levels are found in poor sleepers, with sleep quality assessed objectively via polysomnography, actigraphy, or via subjective measures such as the Pittsburgh Sleep Quality Index (126, 127, 128, 129, 130, 131, 132). ADMA is negatively associated with sleep quality and efficiency and positively associated with sleep latency, a hallmark of insomnia (128), although not consistently across studies (133,134). Most studies primarily involved patients with obstructive sleep apnea rather than psychiatric cohorts, limiting the generalizability of these findings. However, sleep fragmentation from apnea disrupts brain sleep centers, thereby creating a bidirectional link with psychiatric disorders (135).

White matter impairments, macrostructural indicators of brain pathology, commonly affect connectivity and neural circuits early in psychiatric disorders (136). These impairments identified as hyperintense magnetic resonance imaging signals (137), have been associated with clinical symptoms and cognitive performance related to processing speed, memory, attention, and social cognition (138). Although the exact etiology is not known, cerebral small vessel disease, a disorder that affects the brain’s perforating microvessels, is considered a key contributor, with ADMA implicated either through reduced NO production leading to altered cerebral blood flow or through microvascular endothelial dysfunction (139). Systemic elevated ADMA levels are a significant predictor of white matter hyperintensities (40,140, 141, 142, 143). Furthermore, ADMA positively correlates with the severity of white matter hyperintensities (Fazekas score), even after controlling for confounding factors (142,144).

The link between ADMA and transdiagnostic criteria further extends to the molecular level, particularly involving oxidative stress. Systemic ADMA levels positively correlate with oxidative stress and inflammation-associated markers in depression (100,104), alcohol dependence (114), and ADHD (113). In schizophrenia, such correlations are lacking (95,107); however, ADMA negatively correlated with components of the antioxidant defense system, such as vitamin E (95). In line, patients with bipolar disorder during the euthymic phase exhibited reduced ADMA levels compared with healthy control participants (122,123), alongside an increase in antioxidant capacity (123). In cases of sleep deprivation, elevated ADMA levels have been linked to hypoxia (130,131) and NO levels to oxidative stress (132). These findings may reflect a feedback loop where oxidative stress inhibits DDAH1, raising ADMA levels and superoxide via NOS uncoupling. However, excess NO can also trigger nitro-oxidative stress with low ADMA, possibly explaining reduced ADMA as well as elevated oxidative stress in some psychiatric patients (110,116).

ADMA levels respond to therapeutic interventions. Atypical antipsychotic treatment in schizophrenia significantly decreased plasma ADMA to partial (93) or full (96) normalization. Pre- and posttreatment ADMA levels did not correlate with improvements in symptoms as assessed by the standard positive and negative syndrome scales (93). However, treatment effects on ADMA level were associated with improved cognitive functions, specifically in working memory and attention (96). Monotherapy with various typical and atypical antipsychotics, including olanzapine, risperidone, quetiapine, aripiprazole, clozapine, haloperidol, and sulpiride, yielded similar outcomes on ADMA (91,93,96,98). However, clozapine has also been found to increase ADMA levels (106), which is consistent with its well-known adverse effect of inducing myocarditis (145) and ADMA as a recognized marker of cardiovascular risk (21,22). Therapeutic effects of reducing ADMA levels were also found in patients with bipolar disorder receiving lithium (146), in patients with ADHD receiving methylphenidate treatment (113), and in patients following sleep improvement (147). In patients with alcohol dependence, ADMA levels increased following hospitalization and withdrawal, regardless of whether their baseline levels were higher or lower than that of healthy control participants (114,116). While this may be specific to the molecular challenges under withdrawal, ADMA levels did not correlate with the severity of withdrawal symptoms (116). Finally, a number of studies on schizophrenia (107,148), depression (103,108,149), and ADHD (115) do not demonstrate clear effects on ADMA levels, despite observed changes in NO following treatment (115,148,149).

### Genetic Evidence

SNPs in *DDAH1* and *DDAH2* are associated with schizophrenia, bipolar disorder, depression, anxiety, and alcohol dependence, as well as broader traits such as neuroticism, guilt feelings, and sleep quality parameters (12,150). Additionally, *DDAH1* has emerged as a candidate in autism spectrum (12,151) and obsessive-compulsive disorders (151). Genetic variants of *DDAH1* are linked to treatments, such as lithium in patients with bipolar disorder and amphetamine response (12). Beyond clinical symptoms, associations extend to cognitive functions, with *DDAH1* variants linked to visual processing, motor control, attention, executive function, and problem-solving skills, while *DDAH2* variants are associated with attention, logical analysis, and memory retrieval (12). Furthermore, genetic variations intersect with brain morphology, with cortical thickness being associated with *DDAH1* variants (150) and the presence of white matter hyperintensities being associated with *DDAH2* polymorphism (152) (Figure 1, central circle, outer section; Table 2). To our knowledge, no Mendelian randomization studies to date have provided causal evidence.

### Brain Postmortem Evidence

Only a few postmortem investigations have been conducted to inform us whether the systemic alterations extend to the brain (Figure 1, central circle, outer section; Table 3). Proteome analysis revealed reduced DDAH1 expression in the anterior cingulate cortex of patients with schizophrenia, with functional annotation in oxidative stress processes (153). A similar downregulation has also been observed in a transcriptome analysis of the prefrontal cortex in patients with schizophrenia (154). This study found *DDAH1* downregulation only during the early disease stage, linking it to protein interaction networks involved in gene expression, cellular signaling, development, and metabolism. This early change may relate to more severe phenotypes (higher suicide rates) or medication effects. Overall, these findings are consistent with most systemic observations, as the reduction in tissue DDAH1 can explain increases in ADMA and subsequent reductions in NO.

Reduced methylation near the transcription start site of *DDAH2* in the frontal cortex of patients with schizophrenia has been associated with increased gene expression. Subsequent functional enrichment analysis has linked *DDAH2* to metabolic functions, cell signaling, and the regulation of cell death and apoptosis (155). This methylation pattern has also been observed in blood-derived cells, underscoring the relevance of peripheral findings to brain studies (155). However, tissue-specific differences cannot be excluded, as conversely, increased methylation of *DDAH2*’s regulatory regions has been noted in blood-derived cells of patients with schizophrenia, with this methylation status being linked to suicide attempts (156). Increased *DDAH2* expression has been observed in the prefrontal cortex of patients with bipolar disorder, with functional associations with apoptosis (157). A recent functional genomics study identified *DDAH2* as a key gene with pleiotropic effects across psychiatric disorders linked to an allele-specific methylation SNP. This SNP affects promoter methylation, with risk alleles reducing methylation and increasing DDAH2 expression. It has been linked to schizophrenia and depression and possibly to compulsive behaviors, as prominent for Tourette syndrome, anorexia nervosa, and obsessive-compulsive disorder. Although it was first identified in the cerebellum, a similar expression pattern was found in the frontal cortex (158).

Lastly, a recent analysis of expression data in schizophrenia and bipolar disorder found unchanged *DDAH1*/*2* mRNA levels in the prefrontal cortex but significant functional shifts, including disrupted coexpression with psychiatric risk genes and reduced integration into shared gene networks, likely due to altered transcription factor activity (159).

## Building the Case for DDAH Proteins and Their Role in Psychiatric Disorders From Preclinical In Vivo and In Vitro Studies

Although no preclinical model can replicate the exact pathology of psychiatric disorders, such models offer valuable insights into relevant endophenotypes and enable mechanistic exploration (160). Regarding DDAH proteins, current data from preclinical studies can be broadly divided into 2 categories: 1) descriptive changes in DDAH1 or DDAH2 in models relevant to psychiatric disorders and 2) genetic models that manipulate DDAH1 or DDAH2 protein levels, potentially in a global, brain-specific, or cell type–specific manner (Figure 1, central circle, outer section; Table 4).

Rats subjected to maternal separation (161) and mice on a magnesium-restricted diet (162), both of which model depressive-like phenotypes, exhibited reduced DDAH1 levels in the ventral hippocampus, amygdala, and hypothalamus. Consistent with this, chronic peripheral ADMA infusion in rats led to decreased activity commonly associated with depression (163). Anxiety-like phenotypes in rats induced by monosodium L-glutamate treatment also manifested with reduced hippocampal DDAH1 levels (164). Additionally, sleep-deprived rats showed reductions in both DDAH1 and DDAH2, as well as increased ADMA and decreased NO levels in the diencephalon (165,166). Similarly, a genetic model of *Mecp2* null mice, which is relevant for autism-like behaviors, exhibited reduced DDAH1 expression levels in the brain (167). The reported reduction of DDAH1 in the brain of various animal models is consistent with the postmortem findings and systemic increases of ADMA levels in patients across disorders. However, elevated DDAH1 levels have also been reported in animal models exhibiting schizophrenia- or depression-related phenotypes with learning and memory deficits (168,169), as well as in models of sleep deprivation (170,171) and methamphetamine toxicity (172,173). In contrast, other studies, such as those involving morphine tolerance and dependence in mice, have reported no significant effect (174). While informative, these studies lack causal evidence linking DDAH proteins to psychiatric disorders.

Recent attempts to close this gap have utilized genetically engineered models. By comparing *Ddah1* knockout, transgenic, and wild-type mice, DDAH1 was found to promote adult neurogenesis and cognitive recovery after stroke by regulating the hypoxia-inducible factor 1α via NO, which in turn regulates genes involved in acetylcholine-related neurogenesis and repair (175). Additionally, DDAH1 expression increases during nerve growth factor–induced differentiation, thereby accelerating neurite formation without changes in DDAH1 activity and ADMA levels, suggesting ADMA-independent NO regulation (176). Nevertheless, ADMA’s role and systemic effects remain important, as elevated ADMA has been linked to memory deficits (177, 178, 179, 180, 181), blood-brain barrier leakage and neuronal damage (177,178,182,183), and possibly prompting compensatory DDAH1 upregulation in some models (168,169).

DDAH1 further protects dopaminergic neurons of the nigrostriatal pathway from mitochondrial dysfunction and cell death by decreasing intracellular levels of ADMA (184). Rescue experiments suggest that while DDAH1’s regulation of NO can partially explain its protective effects, its role in regulating mitochondrial function may be NO independent (184). These data may provide a mechanistic link between disrupted DDAH1/ADMA axis and striatal dopamine depletion in methamphetamine-treated rats (172). Notably, negative effects of *Ddah1* deletion and the protective benefits of its upregulation at both molecular and behavioral levels were only observed after rotenone injection (184). Consistent with these findings, *Ddah1* knockout mice showed reduced amphetamine sensitivity, indicating dopamine system impairments (185) but minimal behavioral implications, which implies that added stressors may be needed to uncover full effects (178,182).

Despite differing changes in DDAH1 levels across various models, treatments consistently show responsiveness. In instances where DDAH1 levels were reduced (and/or ADMA levels were elevated) in the brain or blood of animal models, treatments successfully restored their expression, either fully or partially (161,162,165, 166, 167,177,179,181). Conversely, when DDAH1 levels were elevated in these models, treatments effectively reduced its expression (169,173). The treatment interventions ranged from clinically used agents (161,162,186) and natural compounds from traditional medicine (165,166,177,179,181) to pharmacological DDAH1 inhibitors (173), neurotransmitter receptor modulators (169), and gene reactivation approaches (167). To date, studies of clinically relevant agents have primarily examined monoamine reuptake inhibitors for treating depressive-like behaviors. Escitalopram selectively increased DDAH1 levels in the hippocampus of maternally separated rats (161). Fluoxetine and venlafaxine increased DDAH1 expression in the hippocampus of healthy rats (186), suggesting nonspecific effects in healthy animals. Finally, paroxetine treatment normalized DDAH1 expression in the hypothalamus and amygdala, reduced oxidative stress, and alleviated depressive-like behaviors, underscoring its potential in treating conditions that involve both depression and oxidative stress (162).

On the other hand, DDAH2 appears to influence cell differentiation. Although silencing *Ddah2* does not directly affect differentiation (176), its expression status serves as a marker for neuronal differentiation (187). A differentially methylated region in the *Ddah2* gene shows hypomethylation in undifferentiated stem cells and hypermethylation in differentiated neuronal cells (187). Conversely, upstream regions are hypermethylated in trophoblast stem cells and hypomethylated in differentiated trophoblast cells, with *Ddah2* expression being repressed under stem cell conditions and increased upon the induction of differentiation (68). These epigenetic patterns are consistent with findings from postmortem brain studies.

## Synthesis and Conclusions

DDAH proteins regulate key targets such as NO, AKT, ERK, and VEGF, which are critical throughout development and involved in psychiatric disorders. DDAH1, the more prominent isoform, shows strong expression and uniquely metabolizes ADMA. ADMA levels are altered in patients with schizophrenia, depression, bipolar disorder, substance use disorders, ADHD, and across transdiagnostic domains. Among the 47 reviewed clinical studies with systemic measurements, 31 (*N* = 8212; mean = 265) found that increased ADMA was linked to psychiatric or related traits. In contrast, 9 studies (*N* = 870; mean = 97) found no changes, and 7 (*N* = 548; mean = 78) reported decreased levels. Studies with positive associations generally had larger, better-powered samples. Diverse diagnoses, frequent comorbidities, varied treatments, inclusion of nonpsychiatric participants, and inconsistent methodology currently preclude a feasible meta-analysis. Furthermore, contradictory results may reflect a nonlinear relationship, with both high and low ADMA levels linked to dysfunction and midrange levels associated with better outcomes (188).

In-line standard treatments tend to normalize both increased and decreased ADMA levels. While DDAH proteins are not yet validated therapeutic targets, emerging DDAH1 inhibitors show promise in other conditions that involve the DDAH1/ADMA pathway. For example, ZST316 reduced vasculogenic mimicry and cell migration in triple-negative breast cancer (189). These findings support further investigation of DDAH1 inhibitors as potential treatments. Moreover, ADMA may represent a diagnostic marker, with additional implications for cognition, white matter changes, and oxidative stress.

Genetic, epigenetic, and expression data reinforce the relevance of both DDAH1 and DDAH2 in psychiatric symptomatology, and animal models generally mirror patient-related changes. However, it is becoming increasingly evident that DDAH1 and DDAH2 have distinct functions. This is also reflected in their differential expression patterns, both in the brain and across other organs (190). However, current research remains imbalanced and skewed toward DDAH1. This should not be misinterpreted as a lack of potential significance for DDAH2, particularly in developmental contexts. To advance the field, large, standardized, longitudinal studies examining both isoforms are needed, ideally through multicenter collaborations. In parallel, isoform-specific cellular, organoid, and animal models will be essential for uncovering causal mechanisms to guide hypothesis-driven research into underlying molecular pathways.
